# Accuracy of Assignment of Atlantic Salmon (*Salmo salar* L.) to Rivers and Regions in Scotland and Northeast England Based on Single Nucleotide Polymorphism (SNP) Markers

**DOI:** 10.1371/journal.pone.0164327

**Published:** 2016-10-10

**Authors:** John Gilbey, Eef Cauwelier, Mark W. Coulson, Lee Stradmeyer, James N. Sampayo, Anja Armstrong, Eric Verspoor, Laura Corrigan, Jonathan Shelley, Stuart Middlemas

**Affiliations:** 1 Marine Scotland Science, Freshwater Fisheries Laboratory, Faskally, Pitlochry, Scotland, United Kingdom, PH16 5LB; 2 Rivers and Fisheries Trusts of Scotland, Suite 1F40, 2 Commercial Street, Edinburgh, Scotland, United Kingdom, EH6 6JA; 3 Environment Agency, Tyneside House, Newcastle Business Park, Newcastle upon Tyne, England, United Kingdom, NE4 7AR; University of Tasmania, AUSTRALIA

## Abstract

Understanding the habitat use patterns of migratory fish, such as Atlantic salmon (*Salmo salar* L.), and the natural and anthropogenic impacts on them, is aided by the ability to identify individuals to their stock of origin. Presented here are the results of an analysis of informative single nucleotide polymorphic (SNP) markers for detecting genetic structuring in Atlantic salmon in Scotland and NE England and their ability to allow accurate genetic stock identification. 3,787 fish from 147 sites covering 27 rivers were screened at 5,568 SNP markers. In order to identify a cost-effective subset of SNPs, they were ranked according to their ability to differentiate between fish from different rivers. A panel of 288 SNPs was used to examine both individual assignments and mixed stock fisheries and eighteen assignment units were defined. The results improved greatly on previously available methods and, for the first time, fish caught in the marine environment can be confidently assigned to geographically coherent units within Scotland and NE England, including individual rivers. As such, this SNP panel has the potential to aid understanding of the various influences acting upon Atlantic salmon on their marine migrations, be they natural environmental variations and/or anthropogenic impacts, such as mixed stock fisheries and interactions with marine power generation installations.

## Introduction

Stock identification in fish species has become an integral component of modern fisheries management and for studying adaptation in wild populations [1, 2]. To manage a species successfully, it is important to understand the underlying structure of the various populations making up the total stock and how exploitation, natural and anthropogenic influences are distributed between the different components [1]. Disregarding this structure has the potential to give rise to misleading conclusions when examining a species’ biological characteristics which, in turn, may lead to differential exploitation of parts of a stock and associated selective changes in phenotypic characters s [3–6]. In extremis, this may impact the viability of individual populations within the total stock [7].

Historically, techniques to identify the origin of salmonids captured away from their natal rivers were based around physical tagging of fish [8, 9]. While such techniques provided invaluable and unambiguous information on the origin of the tagged fish, only relatively small numbers of fish could be studied in this way. Other techniques, such as stable isotope analysis [10], otolith morphology and microchemistry [11], and parasite tracking [12] have also been used to identify stock origins, with varying levels of success.

Advances in DNA profiling and associated analytical techniques has allowed the development of genetic stock identification (GSI) using a number of types of genetic markers [13–15]. Allozymes and mitochondrial DNA have both been successfully used for stock identification in salmonid species [16–18]. Panels of highly polymorphic microsatellite markers have allowed stock identification to be successfully performed with Atlantic salmon at a number of scales, from inter-continental to intra-river [19–22]. In Scotland and the North East of England, the study area of the current analysis, the microsatellite baseline of Gilbey et al. [23] allowed accurate assignment to country, but lacked resolution to allow reliable assignment to river.

Over the last few years, single nucleotide polymorphic (SNP) loci have begun to be available and used in stock identification studies [24–27]. SNPs are among the most common of variations in the genome and recent technological developments in SNP discovery have led to a large number of SNPs being available for use in salmonids [28–30], including in Atlantic salmon [31–33]. Comparisons of the power of randomly selected microsatellites to panels of randomly selected SNPs to define population structure and perform stock identification have shown that both types of markers are likely to be useful in population genetics studies and that a mixed marker approach might be the most effective suite of loci [34, 35]. However, the large number of SNPs available means that optimal combinations of SNPs can be selected, which gives enhanced power in both defining population structure and performing genetic assignments [36]. A major advantage of SNP loci is their compatibility among genotyping platforms and across laboratories means that the sometimes lengthy calibration process required for using microsatellites can be avoided [37].

The Atlantic salmon (*Salmo salar* L.) is an anadromous fish that hatches in freshwater, then migrates to the marine environment before returning to their natal rivers and streams to spawn [38]. This homing behaviour has led to numerous, highly structured, reproductively isolated and locally adapted populations of salmon at a hierarchy of geographic scales [39–41]. Long term conservation of salmon populations is assisted by a greater understanding of their biology and ecology whilst taking into account the different characteristics and status of the numerous populations and stocks [42]. This is especially true due to the marked decline in abundance in many populations over the last few decades [43], which has been associated with a number of factors, including changes in marine mortality rates [44, 45]. Variations in the marine migratory patterns of different salmon populations are known to occur but the full extent of these differences have yet to be resolved [46, 47].

Recent years have seen significant developments in off-shore renewable energy projects (e.g. off shore wind, tide and wave energy devices) in many areas including around the Scottish and English coasts [48]. Environmental impacts of such developments, including those on anadromous species such as the Atlantic salmon, are difficult to quantify but could include negative effects, such as increased noise [49], collisions [50] and interactions with electromagnetic fields [51]. Sustainable management of the development of off-shore renewable energy projects will be greatly aided by an understanding of stock-specific patterns of migration and will allow potential impacts of such developments to be better quantified at the individual stock level [52].

The impacts of such developments have also to be viewed in the context of the much larger scale changes happening in the marine environment associated with climate change. This has the potential to influence the physical and chemical properties of water together with changes in fish, invertebrate and plant species in the freshwater and marine environments [53]. In turn, these responses may give rise to changes in the adaptive landscape the fish are subjected to, which has the potential to influence differentiation and separation of river systems.

The aims of the present study were to investigate genetic structuring in Atlantic salmon in Scotland and NE England using SNP markers and define the resolution that could be obtained for accurately assigning salmon back to their natal rivers or regions. The results of the analysis are discussed in the context of understanding the marine phase of the salmon life cycle and particularly how understanding of the stock-specific impacts of both natural and anthropogenic influences can be understood using the information and techniques presented here.

## Materials and Methods

### Genetic samples

**Fig 1:**
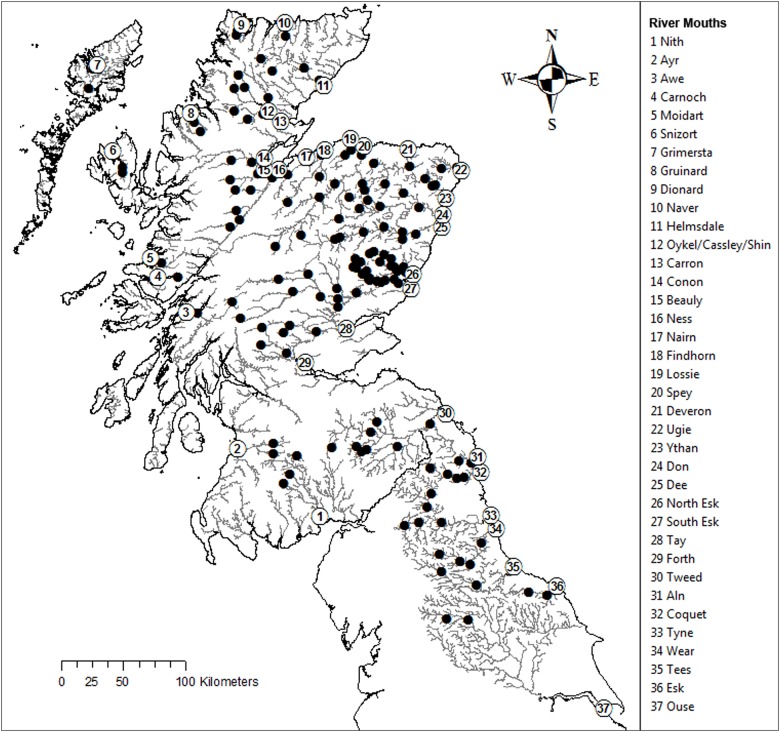
Rivers (numbers) and associated sample collection sites (black circles).

All research carried out for this study was undertaken under UK Home Office regulation by licensed and/or competent personnel. Tissue samples were collected from fish following Standard Operating Procedures agreed with Ethics and Animal Welfare committees at Marine Scotland and the Environment Agency. Fish were collected using electrofishing, fin tissue was collected under anaesthesia (MS222 or Benzocaine) and placed in 100% ethanol, after which the fish was allowed to recover before being returned to the wild. Field permits were granted by Marine Scotland and the Environment Agency. Atlantic salmon fin clips were obtained from 3,787 juvenile Atlantic salmon from Scotland and NE England, originating from 37 rivers and 147 sites. Individual rivers had a mean of four sample sites (minimum 1, maximum 14) with a mean of 26 fish genotyped at each site (minimum 10, maximum 32). Samples represent fish collected between 2002 and 2013 and 1+ parr were preferably targeted A full list of samples sites is detailed in S1 Table and the geographical locations of the sites are shown on Fig 1.

### Sample preparation and removal of full-sibs

Genomic DNA was extracted and purified from individual fin tissue samples using the DNeasy Blood and Tissue purification kit (Qiagen) following the manufacturer’s protocol. Each sample was quantified by fluorometry (Qubit, Life Technologies) and diluted to a concentration of 50ng/μL in TE buffer (10mM Tris-Cl, pH 8.0, 1mM EDTA).

The presence of full-sibs within sites can lead to bias in allele frequency estimates [54] and thus result in potentially misleading outcomes of assignment accuracy determinations. In order to reduce such bias, full sibs were removed from each site, such that a single representative from each family remained for array genotyping. Sibs were identified using the pedigree likelihood approach implemented within the program COLONY2 [55], using either the panel of 15 microsatellites detailed in Olafsson *et al*. [56] (85 sites) or a panel of 96 SNPs (62 sites) (S2 Table for details).

### SNP Array Genotyping

SNP genotyping was carried out at the Centre for Integrative Genetics (CIGENE), Norway. Fish were genotyped at 5,568 SNP loci (for full list see S3 Table) using a modified version of a custom-designed Illumina^®^ iSelect SNP-array [39, 57, 58]. Methods, reagents and protocols are proprietary, but are summarised in Johnston *et al*. [58]. Loci classified as SNP (normal diploid polymorphic SNP) or multi-site variants, MSV-3 (SNP existing on a single paralogue) were retained (see discussion in [59] for details on SNP classifications). All loci with a call rate of < 0.90 were discarded [60].

### Hardy Weinberg Equilibrium

Each sampling site was tested for conformity to Hardy Weinberg equilibrium. For each site and loci combination, Fisher’s exact tests of Hardy Weinberg equilibrium were performed, with the overall measure of equilibrium for a given site being determined using Fisher’s method for combining p-values from independent tests [61, 62]. This was carried out in the *diveRsity* R package [63, 64]. Critical levels of significance were adjusted using the sequential Bonferroni procedure for multiple tests [65].

### Definition of assignment units

**Fig 2:**
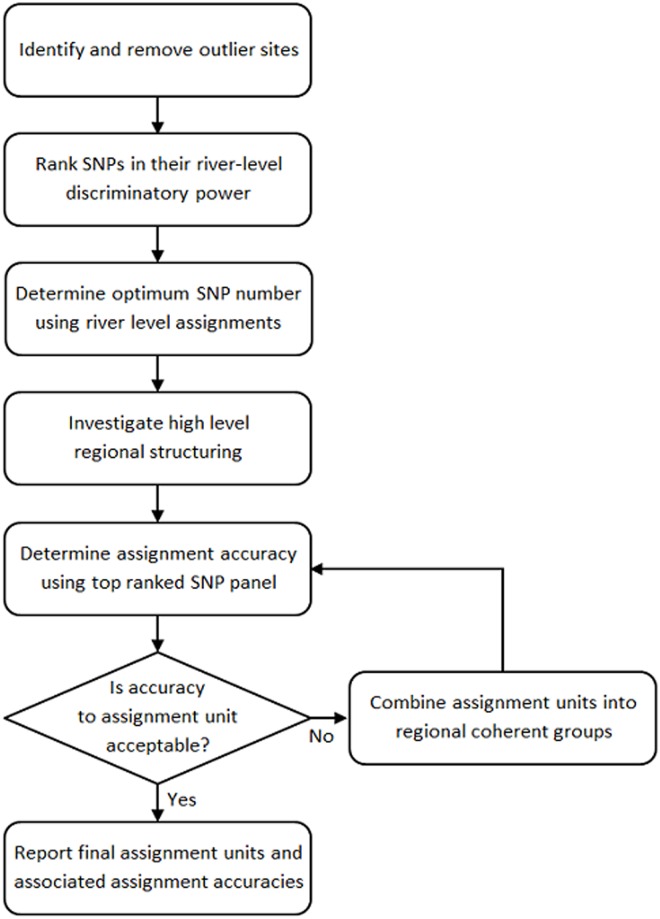
Steps taken in defining assignment units and accuracy of assignment to the units identified.

The aim of the present study was to determine the feasibility of assigning fish back to their natal rivers and, where this was not possible, to define geographically coherent assignment units, based on higher level regional structures containing a number of rivers, to which assignments could be reliably performed. This was achieved using an iterative process as shown in Fig 2.

#### Step 1) Identification of outlier sample sites

In order to identify outlier sample sites, multidimensional scaling (MDS) was carried out using *cmdscale* in R [64], based on estimates of pairwise D_A_ [66] calculated using GenAlEx 6.5 [67]. The presence of such outlier sample sites may influence the identification of a sub-set of the available SNPs for assignment purposes, due to potentially high influence on the various ranking procedures used to evaluate SNP performances [60]. Sites that were seen to be most differentiated from the main clusters of sites on the plots were removed from the initial assignment unit definition and SNP choice (see below) stages of analysis. The outlier sample sites were returned to the dataset after SNP ranking had been performed and included when estimating assignment accuracies.

#### Step 2) SNP choice

In the definition of assignment units and the analysis of assignment success, a sub-set of SNPs were identified that gave maximum power for assignment. SNPs were ranked according to their ability to differentiate between rivers, so that a sub-set of SNPs could be identified and used for further high-throughput screening. However, to avoid ascertainment bias without reducing the power of the analysis, six fish were randomly removed from each site and put into a hold-out set, with the remainder being retained in a training set [68, 69]. The training set was then used to rank and choose the SNPs by calculating F_ST_ [70] for each SNP and ranking the SNP loci according to their discriminatory power at the river level using the R-package HIERFSTAT [64, 71]. Once a ranked list of SNPs was obtained, accuracy of assignment was assessed by assigning fish from the hold-out set back to the training set reference sites using the top ranked 12, 24, 96, 192, 288, 384 and 480 SNPs (the numbers chosen were subsets, or multiples of 96 due to the intention to later use the SNPs identified on a 96 well Fluidigm EP1 platform). Using the hold-out set in this way thus provided relative estimate of likely assignment success using fish not included in the ranking process.

It should be noted that, together with possible ascertainment bias associated with ranking and then testing SNP powers using the same set of fish, which we have tried to avoid by using the approach above, another source of possible ascertainment bias could be due to the origins of the SNP markers making up the panel used. The loci used here were developed from expressed sequence tags using material originating from Norwegian commercial aquaculture strains [39]. However, in the current study we make no inferences on the phylogeographic history of any genetic structuring observed, rather the loci are used as tools for assignment purposes. As such, any potential ascertainment bias associated with SNP origin were irrelevant.

The assignment accuracies of the various SNP panels to river were determined using Bayesian assignment [72] and Monte-Carlo resampling as implemented in GENECLASS2 [73]. This process produces an estimate of the likelihood of individual fish being from each of the assignment groupings examined, with overall assignment accuracy being defined as the proportion of fish *assigned to* a particular unit that have been correctly assigned (i.e. of all the fish assigning to a unit, how many of them are really from that unit). The resampling methods approximate the distribution of genotype likelihoods in the population sampled and then compared the likelihood computed for the to-be-assigned individual to that distribution [74]. The success of assignments was assessed using both the assignments of all hold-out set fish and using a subset of data where only those fish that had been assigned with a likelihood greater than 80%. This exclusion method is similar to that used by Ikediashi *et al*. [75], although summed assignment likelihood scores for all sites in a river were used instead of assignment probabilities at each site. An illustrative cut-off of 80 was used here as an acceptable balance between accuracy of assignments and proportions of fish assigned, but other levels could be used depending on the situation under investigation.

#### Step 3) Regional structuring

The presence of regional structuring in the data was investigated using *k*-means clustering of the full SNP dataset as implemented in the adegenet 1.4–1 R package [76]. Identification of clusters was performed using *k*-means clustering on the results of a principle components analysis of the full dataset. The optimal number of clusters was indicated by an elbow in the curve of Bayesian Information Criterion values as a function of the number of clusters

#### Step 4) Assignment accuracy

Assignment accuracy using the reduced SNP panel was examined using two different techniques. Assignment to rivers, then to assignment regions, was performed by assigning the hold-out set fish to the training set using GENECLASS2, as described above (Hold-out /Training set method). Individual assignment was then also carried out using a Two-fold Cross-Validation approach [77]. Using this approach, all fish were randomly divided into two groups, A and B, each comprising half of the individuals in the total data set. A firstly acted as reference sites with B being assigned to it and vice versa. This technique meant that all individuals were used as both reference and assigned samples. This entire process was repeated 10 times with the mean successful assignment accuracy and variation around the mean calculated over all replicates (N = 20).

#### Step 5) Definition of Assignment units

The creation of the final assignment units was undertaken as an iterative process. Accurate assignments to assignment units were defined by ≥ 80% of fish being accurately assigned. Accuracy was measured using both individual assignment approaches undertaken. If, using both techniques, accuracies to river were over 80%, the river was maintained as the final assignment unit. If both techniques had accuracies below 80% then new assignment units were defined consisting of groups of rivers. These groups were constructed based both on an examination of reciprocal misassignments of fish to and from rivers within the regional structures previously identified and based on the geographical coherence of the units. Finally, if one technique had an accuracy of above 80% but the other had an accuracy below 80%, each river and the assignments to and from it were examined on a case-by-case basis.

Once the final assignment units had been identified, the ranking of the SNPs (Step 2) was undertaken again using these new assignment units and the accuracy of the new SNP panel examined and compared to the original one.

#### Step 6) Final assignment accuracy

Once both the SNP panel and assignment units had been identified, assignment accuracy was examined using the two different techniques outlined above (Step 4) and also by mixed stock analysis. Mixed stock analysis was examined in the software package ONCOR [78] using both 100% single assignment unit sample simulations (where mixtures of fish from each single assignment unit are simulated separately and assigned back to the full reference assignment units) and more realistic fishery mixtures containing fish from each assignment unit. Mixed stock accuracy was assessed using a maximum-likelihood approach where genotype frequencies for each locus in each population were re-sampled using the method of Anderson *et al*. [79] to simulate mixture genotypes and to estimate their probability of occurring in the samples. 100% simulations were based on 1000 simulations of 200 fish per reporting unit and the same simulated reference sample sizes as in the actual dataset. ‘Realistic’ fishery mixtures were based on 1000 simulations of 1000 fishery samples, again using the same simulated reference sample sizes as in the actual data. Simulations were performed using two simulated fishery mixtures, firstly a mixture with equal numbers of fish from each of the assignment units identified, and secondly one in which the proportions of fish were based on the reported rod catch returns [80, 81].

### Loci under selection

The primary aim of the development of the panel of genetic markers described here was to maximise levels of accurate assignments to reference assignment units with maximum levels of resolution within the areas covered. As such, all loci were used in the development of the final panel and it is this panel which is presented in the main body of the manuscript. It is important to remember, however that although the inclusion of F_ST_ outlier loci potentially under selection may benefit assignment resolution and accuracy, this may also result in contrasting genetic structure being identified in comparison to neutral markers alone. Furthermore, a number of approaches and software packages (for example the popular STRUCTURE [82] package) used for the examination of population structure and associated techniques of assignment, have assumptions that rely on the neutrality of the markers used. Although these packages were not used here, it is important that confusion is avoided in the future if analysis of this reference dataset is to be performed using such techniques.

To avoid such confusion, a second analysis was performed. This followed exactly the techniques outlined above, the difference being that before starting the development of the assignment units, outlier loci were firstly identified and then removed from the dataset. Development of assignment units and testing of assignment accuracy then proceeded as described. Analysis of outlier loci were conducted using the two software packages BayeScan v2.01 [83] and OutFlank [84]. Using default settings in both packages, loci identified as outliers by either package were removed from the dataset.

As the main aim of the analysis presented here was to maximise assignment power in order to aid management applications, the results described in the main body of the manuscript are those containing the full set of markers. Results from the outlier tests and the full analysis using the neutral markers only are described in S1 Dataset.

## Results

Quality control of the SNP types identified 709 MSV-3 and 3,715 SNP markers that all had call rates > 0.90, giving a panel of 4,424 SNP markers (for full list see S3 Table). After correction for multiple tests, a single site was found to be out of Hardy Weinberg equilibrium (Upper Cassley in the Kyle system). This site was also identified as an outlier (see below) and so was removed from the ranking analysis.

### Identification of outlier sample sites

**Fig 3:**
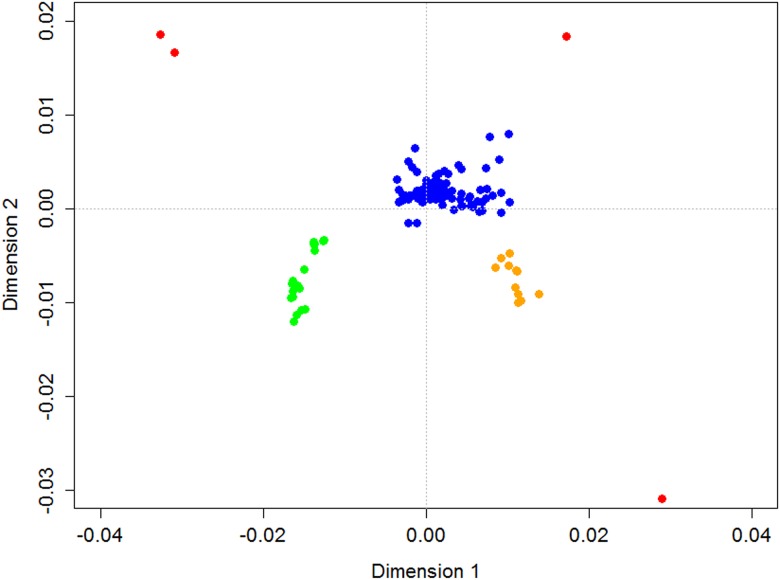
Multidimensional plot of pairwise D_A_ based on all SNPs. Red points are sites identified as outliers; green points are sites south of the Tweed on the East coast; orange points are Kyle of Sutherland sites (i.e. around sites 12 and 13 on Fig 1) and blue points represent the remaining Scottish samples.

Examination of the MDS plot identified four outlier sample sites: one each from the rivers Orchy and Cassley and two from the Ouse (Fig 3). Regional structuring is already apparent on this plot with sites south of the Tweed (river 30, Fig 1) and sites within the Kyle of Sutherland (rivers 12, 13, Fig 1) region showing clear separation in this analysis.

### SNP choice

**Fig 4:**
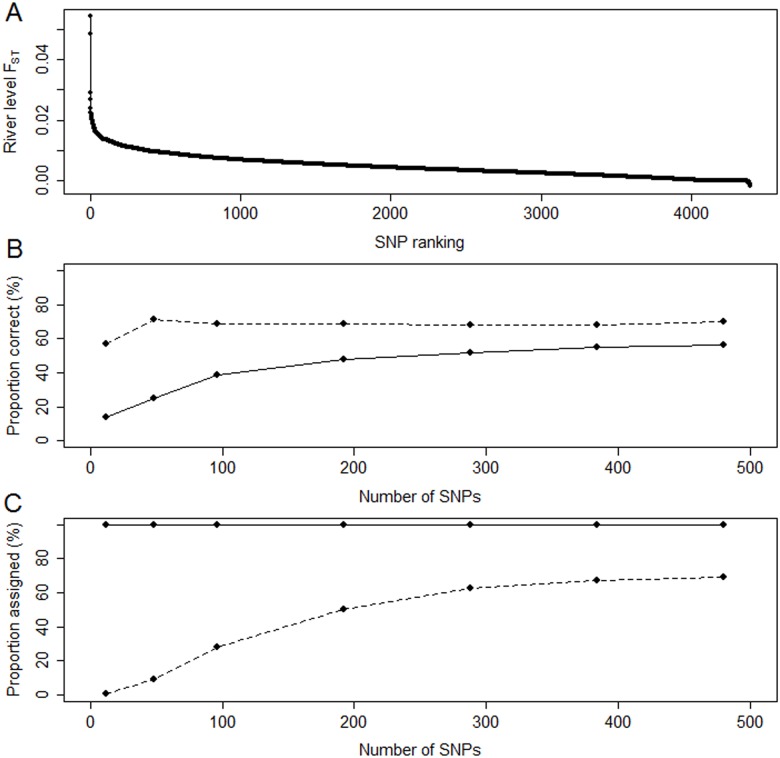
SNP ranking and assignment accuracy to river. A) between-river F_ST_ values for the ranked SNPs. B) assignment accuracy of hold-out set fish assigned to training set reference sites using different numbers of the top ranked SNPs. C) proportion of hold-out set fish assigned to training set reference sites using different numbers of the top ranked SNPs. Solid lines represent all fish, dashed lines only those fish with an assignment likelihood score of at least 80.

Ranking of the SNPs according to their river-level F_ST_ values resulted in an exponential decay pattern of discriminatory power (Fig 4A). MSV-3 SNPs had, on average, significantly higher ranking positions than regular SNP loci (Kruskal-Wallis chi-squared = 226.2, df = 2, p-value < 0.01) with MSV-3 mean ranking being 2068.7 and SNP mean ranking being 2218.5 (median 2057 and 2225, respectively).

When assigning fish to river using different numbers of the top ranked SNPs, the accuracy of the assignments increased in an asymptotic manner, with accuracy levelling off above 288 SNPs (Fig 4B). If the assignment likelihood cut-off score of 80 is used, the accuracy of assignment remained relatively constant across all SNP number examined. However, as the number of SNPs increase, so too did the number of fish remaining in the analysis (Fig 4C). Again, this increase was asymptotic. Taking into consideration both the accuracy of assignments and the number of fish assigned when an assignment likelihood score cut-off is used, it was decided to focus on a panel of 288 markers for further analysis (full list of the 288 panel in S3 Table). There was little difference in the patterns produced in further analysis using all fish compared to using an assignment score > 80. Therefore, we present the results based on using an assignment cut-off of 80, with the results for all fish available in the S4 and S5 Tables.

### Regional structuring

**Fig 5:**
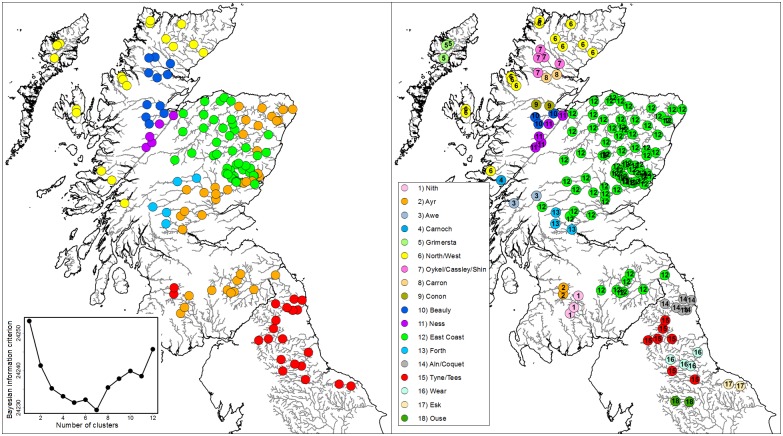
Assignment units as defined by *k*-means clustering. A) Results of *k*-means clustering (insert) showing *k* = 7 as best cluster number, shown by differently coloured symbols. B) Final assignment units after combining rivers with reciprocal misassignments.

The results of the clustering analysis suggested the presence of seven clusters which show generally good coherence with geographic position, with some discontinuity (Fig 5A). Cluster 1 is comprised mainly of English sites with 2 sites from SW Scotland; cluster 2 is focused around the south of Scotland (both coasts) and around the rivers Forth and Tay with some sites also along the East coast. Cluster 3 represented exclusively NE Scottish sites while 4 comprised sites on the Ness system. Cluster 5 represented the Conon, Carron and Oykel/Cassley/Shin sites, cluster 6 sites from the North and West of Scotland and cluster 7 sites from the upper Tay and Forth.

### Definition of assignment units

The proportion of accurate assignments to river using the hold-out/training set approach varied greatly between different rivers; from 100% in nine cases to 0% in two others (Table 1. For full breakdown of assignments see S4 Table). For 12 out of the 37 individual rivers both assignment techniques achieved above 80% accuracy (Table 1) and so they were retained as separate assignment units. There were 22 rivers where neither of the techniques achieved over 80% accuracy. Among these, examination of reciprocal misassignments, geographic location, and regional groups identified by the K-means clustering analysis, resulted in a total of six assignment units into which various numbers of geographically close rivers were combined (Table 1; S4 Table). For the remaining 3 rivers, Grimersta, Carron and Tyne, the hold-out/training set had above 80% accuracy and the Two-fold Cross-Validation was less than 80%. The Grimersta is located on the west coast on the Isle of Lewis in the Outer Hebrides and had 100% accuracy with the Hold-out/training set and very close to 80% with the Two-fold Cross-Validation approach (79.4%). Taking into consideration both the geographic separation of this site from the rest of the sites and the accuracies obtained, it was decided to retain the Grimersta as a separate assignment unit. The Carron is located in the Kyle of Sutherland fisheries area, immediately south of the Oykel/Cassley/Shin system and north of the Conon, both of which were retained as separate assignment units based on the accuracies obtained. Taking into account geographic position, accuracy to neighbouring rivers, the 100% accurate hold-out/training set accuracies, and the 75.8% two-fold cross validation results, it was decided to retain the Carron as a separate assignment unit. The final river, the Tyne,was combined with the Tees, which had accuracies below 80%, to make a joint assignment unit. *Final assignment accuracy and assignment units*

**Table 1:** Assignment accuracy to river of hold-out set fish to training set reference sites using the top ranked 288 SNPs.

Assignment units—River	Sample size	Proportion assigned	Proportion correctly assigned	Assignment units	Proportion assigned	Proportion correctly assigned	Assignment units	Proportion assigned	Proportion correctly assigned
Nith	92 (18)	83.3/77.9	**100/97.6**	Nith	83.3/78.6	**100/97.4**	Nith	88.9/79.4	**100/97.4**
Ayr	64 (12)	100/81.4	**100/97.2**	Ayr	100/81.9	**100/97.2**	Ayr	100/82.2	**100/97.2**
Awe	64 (12)	100/96.6	**100/99.7**	Awe	100/96.8	**100/99.7**	Awe	100/96.8	**100/99.7**
Carnoch	32 (6)	100/71.7	**100/91.3**	Carnoch	100/72	**100/91.3**	Carnoch	100/72.7	**100/91.3**
Grimersta	60 (18)	50.0/66.5	**100**/79.4	Grimersta	61.1/67.7	**100**/78.6	Grimersta	66.7/69.5	**100**/78.6
Moidart	31 (6)	75/64.3	40.0/74.3	West	75/64.5	75.0/70.6	North & West	88.5/66.3	**85.1/80.7**
Snizort	64 (12)	66.7/64.8	75.0/70.2						
Gruinard	62 (18)	55.6/58.6	66.7/58						
Dionard	38 (12)	33.3/60.8	0/60.9	North	71.4/63.7	70.0/66.1			
Naver	95 (18)	55.6/62.8	70.0/57.4						
Helmsdale	64 (12)	66.7/55.4	41.7/50.9						
Oykel/Cassley/Shin	157 (30)	100/84.4	**93.6/87.9**	Oykel/Cassley/Shin	100/84.4	**93.6/89.0**	Oykel/Cassley/Shin	100/84.4	**93.5/89.0**
Carron	64 (12)	83.3/77.1	**100**/76.3	Carron	83.3/77.1	**100**/75.8	Carron	83.3/77.1	**100**/75.8
Conon	64 (12)	100/85.1	**84.6/90.3**	Conon	100/85.1	**84.6/90.0**	Conon	100/85.3	**84.6/90.0**
Beauly	72 (18)	94.4/79.5	**100/94.7**	Beauly	94.4/79.5	**100/94.2**	Beauly	94.4/79.5	**100/94.2**
Ness	120 (30)	93.3/81.8	**96.2/99.3**	Ness	93.3/82.3	**96.3/99.4**	Ness	93.3/83	**96.2/99.4**
Nairn	24 (6)	33.3/37.9	0/3.8	North East	87.1/63.6	**87.8/85.8**	East Coast	95.3/68.4	**94.9/92.8**
Findhorn	71 (18)	61.1/50.1	66.7/70.7						
Lossie	48 (12)	66.7/42.8	28.6/20.6						
Spey	253 (48)	45.8/47.8	25.7/29.6						
Deveron	144 (30)	36.7/46.9	38.5/21.7						
Ugie	39 (12)	33.3/49.9	50.0/79.8						
Ythan	65 (18)	44.4/47.4	33.3/36.3						
Don	87 (24)	41.7/42.3	55.6/22.4						
Dee	252 (48)	47.9/51.3	37.5/40.5						
North Esk	232 (72)	45.8/46.4	46.2/25.5						
South Esk	333 (84)	41.7/46.1	35.9/33.9						
Tay	296 (72)	59.7/57.1	69.8/65.5	Tay/Tweed	77.5/62.9	69.5/73.3			
Tweed	226 (48)	58.3/56.8	63.2/62.6						
Forth	70 (18)	88.9/67.9	**84.6/89.9**	Forth	94.4/72.3	**84.6/90.0**	Forth	100/76.0	**84.6/90.0**
Aln	48 (12)	50.0/63.5	37.5/39.6	Aln/Coquet	84.4/66.7	**92.3/85.0**	Aln/Coquet	87.5/68.3	**92.3/85.0**
Coquet	96 (24)	70.8/63.1	73.3/62.9						
Tyne	120 (30)	93.3/70.6	**84.6**/76.7	Tyne/Tees	90.5/72.3	**87.2/91.7**	Tyne/Tees	90.5/72.3	**87.2/91.7**
Tees	48 (12)	50.0/68	42.9/53.8						
Wear	96 (24)	87.5/73.8	**87/92.7**	Wear	87.5/74.6	**87.0/92.8**	Wear	87.5/74.8	**87.0/92.8**
Esk	48 (12)	83.3/66.8	**100/83.4**	Esk	91.7/68.8	**100/83.5**	Esk	91.7/70.0	**100/83.5**
Ouse	48 (12)	100/87.4	**100/100**	Ouse	100/87.4	**100/100**	Ouse	100/87.4	**100/100**

Values to the left of the / separator represent results using the removal method, values to the right of the / separator represent results using the Two Fold Cross Validation method. Values are from only those fish with assignment likelihood scores of 80 or above. Assignment accuracy is shown firstly for all rivers, then for assignment units comprising groups of rivers. Values in bold represent accuracy of at least 80% of correctly assigned fish to particular units. Columns 1–4 represent assignment at the initial river level, with columns 5–7 and 8–10 representing assignments to assignment units containing groups of rivers/units which have been grouped iteratively. Sample size is number of fish in each river with those removed from this total and retained as Hold-out set in parenthesis.

The new Aln/Coquet, Tyne/Tees and North East combined units showed assignment accuracies above 80% for both techniques. However, there were three cases where the new assignment units had assignment accuracies ~ 70%; the West, North and Tay/Tweed untis. In order to increase accuracy, a final stage of combination was performed with the West and North units being combined and the Tay/Tweed combined with the North East unit. This resulted in accuracies to the new combined units of above 80% with both techniques (Table 1). In addition to the proportion of accurate assignments to the new combined assignment units being greater than to individual rivers, the proportion of fish assigned to each unit also increased (Table 1. For full breakdown of assignments see S5 Table).

### Mixed-stock fishery simulations

**Fig 6:**
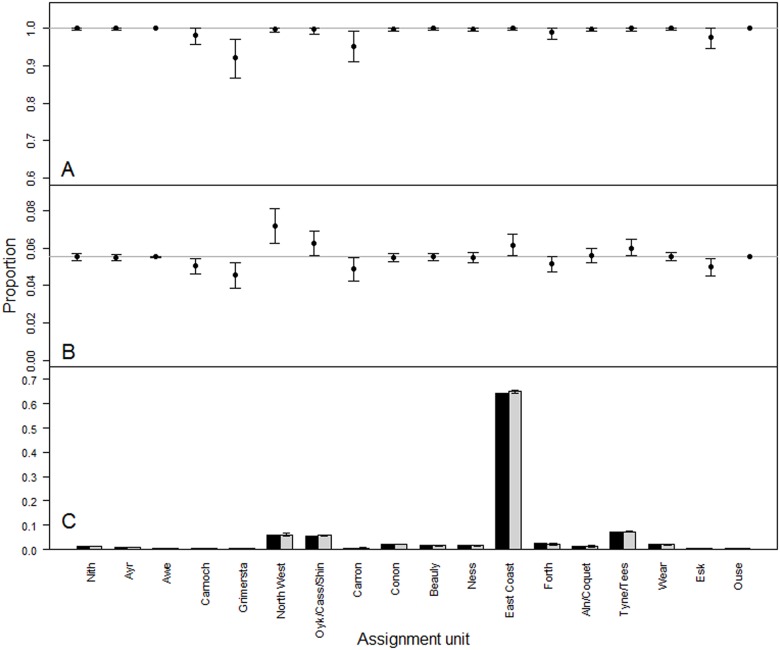
Results of the fishery simulations of the assignment units identified using the top ranked 288 SNPs. A) 100% simulations, B) Mixed Stock Fishery simulation with equal proportions in each assignment unit, C) Mixed Stock Fishery simulation with proportions in each assignment unit based on productivity of that unit. In A and B, horizontal grey lines represent actual simulated proportions and points represent mean simulation proportion estimates. In C, black bars represent simulated proportions and light bars mean simulation proportion estimates. In all plots, bars represent 95% confidence intervals calculated over 1000 replicate simulations.

The results of the various mixed stock analysis fishery simulations are shown in Fig 6. It can be seen that, in most situations, the estimated proportions matched well with the actual proportions used in the simulations. Fig 6A shows the combined results of the 18 individual 100% simulations. In 16 out of the 18 assignment units, the estimated proportions contained the simulated proportions. The confidence intervals (CI) of the Grimersta (CI 0.868–0.970) and Carron (CI 0.910–0.993) did not encompass the actual proportions of fish simulated, with the largest difference between the upper confidence interval and the true simulated value being 0.03 in the Grimersta.

For the equal mixed stock fishery simulation (Fig 6B), again, estimated proportions matched well with the actual proportions used in the simulations, and the difference between the estimated and actual values was small. Furthermore, a significant increase in accuracy is seen using the combined assignment units as compared to the initial individual rivers (see S1 Fig). The units Carnoch, Grimersta, Carron, Forth and Esk had slight underestimations (max difference between upper CI and true proportion 0.004), and the North & West had a small overestimation (difference between lower CI and true proportion 0.007).

The final mixed stock analysis using different simulated proportions of fish based on the reported rod catches was accurate in all cases, with the confidence intervals of all the estimates encompassing the actual simulated proportions of fish (Fig 6C). As these simulations used different proportions of stocks, the standard error (SE) of the mean was also calculated. Two assignment units had SEs that did not encompass the true simulated proportions. The Forth, with a simulated proportion of 0.027 had an estimated proportion of 0.023 (SE interval 0.021–0.025) and the East Coast unit had a simulated proportion of 0.643 and an estimated proportion of 0.650 (SE interval 0.646–0.653). The difference between the actual and the standard error intervals were thus 0.002 and 0.003, respectively.

### Neutral Loci analysis

Results from the outlier analysis and subsequent assignment unit and assignment accuracy analysis are contained in S1 Dataset. From the initial 4425 SNPs, 457 and 59 outlier SNPs were identified by BayeScan and OutFlank, respectively, with 48 of these being in common. As such a total of 469 SNPs identified as outliers by either or both of the techniques were removed from the dataset, leaving 2956 SNPs available for analysis. From the panel of 288 SNPs identified above using the full SNP set, 85 were classified as outliers. Results from the full analysis of assignment units and assignment accuracy with the neutral-only SNP set are detailed in S1 Dataset. It can be seen that, to obtain assignment accuracies on a parr with the final SNP panel containing all SNPs, a significant loss of assignment unit resolution is required. The full panel has 18 assignment units, with crucially the East coast being separate from the North and West coasts, whereas the neutral-only SNP panel had these units combined. So while assignment accuracy is generally maintained using neutral-only markers, the resolution has been significantly reduced, which, in turn, has implications for utilisation of the panel as a management tool.

## Discussion

Accurate, reliable and cost-effective techniques of performing genetic stock identification are important in helping to provide an understanding into the migratory patterns of the various components making up the total salmon stock [85]. Such information is useful in understanding the impacts of natural or anthropogenic changes in the marine environment through mechanisms such as climate change, mixed stock fisheries and offshore developments associated with energy generation. The results of the work presented here confirm the utility of SNP markers for performing GSI with Atlantic salmon and highlight the level of assignments that are currently possible. Accurate assignments are seen to be possible to the river-level in a number of cases, or to regionally coherent assignment units when river-level assignments proved problematic. The definition of assignment units is partly dependent on the situation under investigation and the trade-off between the geographic resolution required and the level of certainty attached to the assignments. For example, if geographic resolution was the most important factor using units defined with a 70% cut-off may be appropriate whereas, if certainty of assignment was more important, than geographic resolution units based on the 80% cut-off would be more appropriate (Table 1). Whichever approach is taken, the genetic baseline and approach to defining and examining units presented here should provide a useful resource for helping to understand the migratory marine phase of the salmon’s life history.

Such an understanding is of particular interest in the present situation the species finds itself in. Global changes in temperature and associated oceanic conditions can impact growth and survival of different stocks, depending on their migration routes and feeding areas [86, 87]. Identification of the natal origin of fish in the marine environment thus has the potential to greatly benefit the understanding of stock-specific patterns of oceanic utilisation. More local developments, such as marine renewable devices also have the potential to impact on the salmon’s migratory patterns [50–52]. Relatively little is known about the migratory routs of Scottish salmon upon return to the UK coastline. Conventional tagging suggests fish do not migrate directly to their natal rivers, but rather spend a period of time migrating around the coast, with fish tagged in a particular location appearing throughout the country [88]. Again, an ability to utilise all fish as being genetically, rather than physically, tagged has the potential to greatly enhance the ability to better understand coastal migration in the face of continuing development in this area.

It is of note that the panel of 288 SNP loci identified here contained MSV loci. In many studies, such loci are filtered out, often due to the difficulty of genotyping such loci on many platforms [89, 90]. Whole-genome duplications and associated MSV’s may be found throughout the genome and may facilitate adaptation through neo-functionalisation or increased gene expression [91]. Removal of such loci, therefore, has the potential to impoverish the potential power and interpretation of genomic analytical studies, as signals from such loci are ignored [92]. In turn, this may have an impact on assignment accuracies. Although the direct influence of incorporating MSV3 was not examined here, it is interesting to observe that the 288 SNP panel of highest ranked loci contained 20.1% MSV loci compared to 16% in the dataset as a whole, with mean ranks within this panel of 129.5 and 148.3 for the two marker types, respectively. These observations suggest that, as predicted, the MSV3 loci facilitate enhanced assignment power. It does not appear, however, that the MSV3 loci are overrepresented in the loci identified as being under selection, with just 17.1% of these loci being MSV3 compared to the 16% in the full dataset.

The various methods utilised here in defining the assignment units and then testing the accuracy of these units acted in an iterative way. Assignment were first made to river and then rivers combined into assignment units based on information from the misassignments between neighbouring rivers and the regional analysis. We suggest that this, together with the different techniques used to test these assignment units, provides a robust approach to defining both units and expected assignment accuracies. It is well known that methods of testing assignment accuracy may suffer from bias, such as sampling bias, ascertainment bias, and a lack of cross-validation [68, 69]. Here, we used both ‘blind’ samples of fish removed from the dataset before SNPs have been ranked and panels determined, together with two-fold cross validation to examine individual assignment, and both 100% and realistic fishery simulations to examine mixed stock analysis. The broadly similar estimate of accuracy obtained with all techniques provides confidence in these estimates.

In order test the accuracy of assignments and avoid ascertainment bias fish not included in the SNP ranking procedure should be examined. This was performed here by removing six fish from each site before ranking was performed. Although this number might seem small, the large number of sites represented meant that this resulted in a total of 882 test fish. Furthermore, as the final assignment units represented both rivers and groups of rivers the actual numbers of these ‘blind’ test fish per final assignment unit increased significantly. For example, the two largest assignment units of the East Coast and North & West had 492 and 78 test fish, respectively and the remaining assignment units a mean number of 21.2 each (median 18, inter-quartile range 12–25.5).

The resolutions of the previously available microsatellite-based genetic baselines covering the study area, although useful, were limited compared to that achieved here. The baseline of Griffiths et al. [19] covered the west of Scotland and managed to reliably assign fish to two large regional units covering central Scotland/eastern Ireland and northern England/the borders of Scotland. The baseline of Anon et al. [93] split the study area into three units comprising mainly north and west coasts of Scotland and Ireland, sites surrounding the Irish Sea and sites from the east and central parts of Scotland. However, there was considerable overlap of the boundaries of the units and some units stretched across different coasts of Scotland. The assignment units identified here, using the SNP markers, had higher resolution and geographic coherence and, as such, represented a step forward in the ability to identify the natal origin of salmon.

Enhanced resolution compared to previous genetic coverage was achieved using the SNP markers utilised here. In a number of cases the discriminatory power at the river-level proved very good. However, in other cases, particularly along the East coast, river-level assignments proved impossible. Here, assignment units were defined covering a number of the biggest producing rivers in the area [94]. As a number of these rivers are the most important in the area, of which some are classed as special areas of conservation for salmon, it is unfortunate that it did not prove possible to reliably assign fish to these individual rivers. It may be possible, in the future, to improve levels of differentiation between these rivers by increasing sample sizes and/or sampling numbers.

It should be noted that, in areas where coverage of the individual rivers is comprehensive and where such rivers have been retained as separate assignment units within these regions, future assignments to these individual rivers might be expected to be robust. However, in others areas, where coverage is not so comprehensive, the individual river units as defined here may encompass some of the neighbouring rivers not sampled. For example, the river Nith is represented in the SNP coverage (Assignment unit 1 on Fig 5B) but other rivers from the surrounding area are not represented. Future assignments using the SNP data as presented here will have to take the coverage into account and analysis that result in fish being assigned to, for example, the Nith should be treated as Nith *area* until further assignment unit boundary definition has been performed. The observation holds for all river-level assignments performed for rivers on the west coast south of the West assignment unit.

The ability to distinguish between and accurately assign fish to adjacent rivers in some parts of the study area but not in others has been found in other studies of Atlantic salmon. Palstra et al. [95] found low or absent levels of differentiation in some areas of the Newfoundland/Labrador region and relatively high levels in others. Wennevik et al. [96] found a similar pattern between rivers in Northern Europe and Griffiths et al [19] found differing levels in the north (Ireland, northern England and western Scotland) compared to the south (Spain, northwest France and southern England) of their study area. The various forces involved in determining patterns of genetic differentiation within and among populations are complex and include interactions between evolutionary and contemporary levels of gene flow [95]. These, in turn, have and continue to be mediated by numerous factors including past geological events, founder effects, levels of straying, population sizes, selective pressures, landscape features and environmental and life-history variations [97, 98]. It is unclear from the present study which of these factors may have influenced the patterns of genetic variation seen at the markers used within the study area, and further analysis is required to address this question.

Of course, as is the case with any panel of genetic markers, the origin of the panel has the potential to influence the levels of resolution and assignment accuracy obtained. Such ascertainment bias could result in actual differences between areas being present but not being able to be detected using the SNPs available. This observation does not invalidate the findings presented here, but rather suggests that enhanced resolution may be possible with other markers and so further investigations are perhaps merited to try to split some of the larger assignment units defined.

It is interesting to compare the levels of resolution associated with accurate assignments achieved when using all SNP markers with that when using just neutral markers. The aims of the study presented here were very much to maximise resolution and so aid in management-related questions involving determination of the natal origin of salmon around the Scottish coast. For example, the development of marine renewable energy sources around the coast has the potential to impact migratory routes of salmon and understanding migratory patterns has been identified as a research priority [88]. Assignment unit resolution using all markers was sufficient to be able to separate fish from the North & West and East coasts of Scotland whereas this was not able to be achieved when outlier loci had been removed and, as such, would be preferred when maximum levels of resolution are required and assumptions of neutrality can be ignored. However, in other situations, for example studies into the phylogeographic population structures and/or analysis and assignments using techniques which assume marker neutrality, the set of neutral markers should be utilised. As with any marker panel therefore, care must be taken in future analysis to use a panel whose origin is known and which does not break any assumptions made during such investigations.

Accurate between-river genetic stock identification within the assignments units, as defined in the current study, will require further investigation. However, the SNP structuring as described provides a useful tool for fishery managers. For the first time, fish caught in the marine environment can be confidently assigned to geographically coherent units within Scotland and NE England, including a number of individual rivers. As such, the resource has the potential to aid understanding of the various influences acting upon Atlantic salmon on their marine migrations, be they natural environmental variations and/or anthropogenic impacts, such as mixed stock fisheries and interactions with marine power generation installations.

## Data Accessibility

Data Availability: The authors confirm that all data underlying the findings are fully available without restriction. The raw genotypes for the baseline SNP panel been deposited to Dryad (http://dx.doi.org/10.5061/dryad.12d36).

## Supporting Information

S1 Dataset Full details of identification of outlier loci and analysis on dataset with these removed. (XLSX)

S1 Fig Results of the fishery simulations to river using the top ranked 288 SNPs. (DOCX)

S1 Table Sample sites contained in the baseline and numbers of fish at each site. (XLSX)

S2 Table SNPs used in sib screening. (XLSX)

S3 Table SNP information for full screening and final panel. (XLSX)

S4 Table Assignment accuracies to river using top 288 SNPs and both HS/TS and TFCV techniques. (XLSX)

S5 Table Assignment accuracies to final assignment units using top 288 SNPs and both HS/TS and TFCV techniques. (XLSX)
