# Supplementary material for: Accuracy of Assignment of Atlantic Salmon (Salmo salar L.) to Rivers and Regions in Scotland and Northeast England Based on Single Nucleotide Polymorphism (SNP) Markers
Source: PLoS One. 2016 Oct 10;11(10):e0164327. doi: 10.1371/journal.pone.0164327 (PMC5056707; doi:10.1371/journal.pone.0164327)
Supplement: S1 Fig — (DOCX) [file pone.0164327.s002.docx]

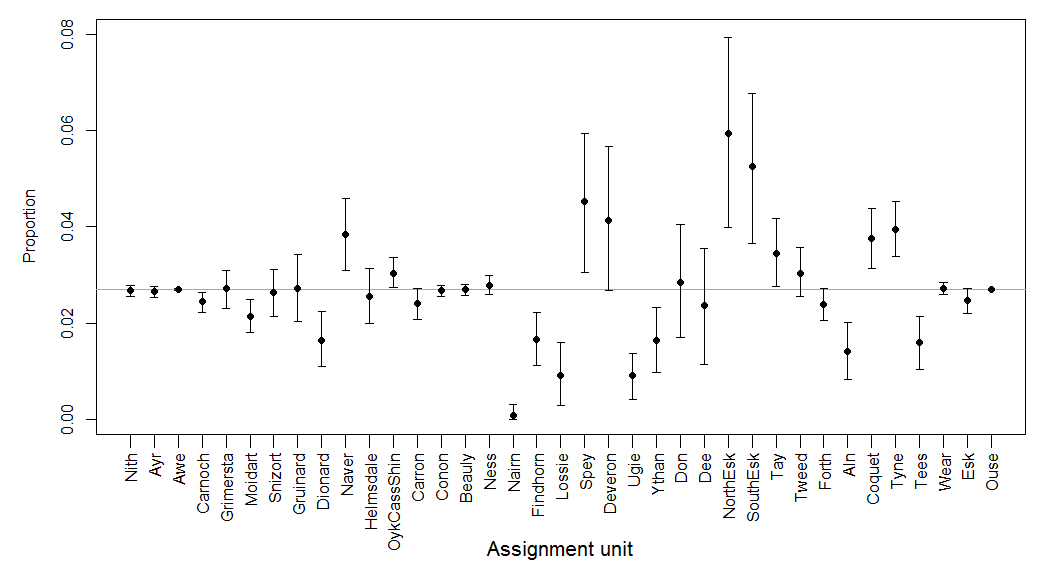


Supplementary Figure 1. Results of the fishery simulations to river using the top ranked 288 SNPs. Horizontal grey lines represent actual simulated proportions. Points represent mean simulation proportion estimates and bars ± 95% Confidence Intervals on these estimates.
